# Items

**Published:** 1906-03

**Authors:** 


### ITEMS.

---

The J. W. Evenden Company, 18 East Seneca Street, Buffalo, invite attention to their wines and liquors for medicinal purposes. In our advertising columns will be found a special offer for trial purposes which physicians will do well to note.

---

Apollinaris is still the "Queen of Table Waters," notwithstanding the many rivals that seek to supplant it. It is the most agreeable to the taste of all the carbonated waters and has a wider range of use than any other potable water. In sickness or in health it is the water, par excellence, for the table, the club, or the traveler. Apenta, the Natural Hungarian Aperient water, also should be remembered by every physician in prescribing a laxative.

---

The Lambert Pharmacal Company of St. Louis, was given the highest award—the gold medal—at the Lewis and Clark Centennial Exposition held at Portland, Oregon in 1905, for their exhibit of "Listerine," and "Listerine Dermatic Soap." This excellent house enjoys the confidence of the medical profession in all parts of the world wherever their products are known.

The Arlington Chemical Company, issued for February as a contribution to their portfolios of historic medical characters, a portrait of Hippocrates, which is a photogravure in colors, of considerable artistic merit.

---

The Mellins Food, of Boston, won the highest award in its class —the gold medal—at the Lewis and Clark Exposition held at Portland, Oregon last year. At the Louisana Purchase Exposition at St. Louis in 1904, Mellin's Food was also awarded the Grand Prize, likewise the highest award. These are substantial recognitions of merit and bespeak continued confidence in this invaluable infants food.

---

The H. K. Mulford Company, Philadelphia, has issued a handsome brochure entitled "Antistreptococcic Serum; its value in Surgery and Medicine with Special Reference to its Prophylactic Use." It is handsomely illustrated, giving views of the plant where the serum is prepared and of pathologic appearances of diptheria in colors.

---

Battle & Company, of Saint Louis, have just issued the ninth of the series of twelve illustrations, of the intestinal parasites, which they will send free, to physicians on application.

---

The vigilant physician finds quite as many uses for Antiphlogistine in the spring as in the winter. In many sections of the world where *The Bloodless Phlebotomist* circulates, sporadic cases of pneumonia will be seen for some time to come. Indeed, pneumonia seems to be like the poor, as we have it always with us, figuratively speaking. The man who treats this disease without covering the thoracic walls, front, sides and back with Antiphlogistine, loses an assistant of the greatest ability. Pleurisy will also be seen for a considerable period, and here Antiphlogistine works to equal advantage.

